# The mechanism of the Shuang Bai Su Qing recipe in treating periodontitis based on network pharmacology and molecular docking technology

**DOI:** 10.1097/MD.0000000000035139

**Published:** 2023-11-03

**Authors:** Haomin Zhang, Ruirui Ning, Yongqin Zhao, Zhao Yan, Yongzhi Chen

**Affiliations:** a Changchun University of Chinese Medicine, Changchun, China.

**Keywords:** Flavonoids, *Houttuynia cordata*, MAPK signaling pathway, molecular docking, network pharmacology, periodontitis

## Abstract

**Aim:**

To explore the material basis of action of the Shuang Bai Su Qing recipe in the treatment of periodontitis using network pharmacology.

**Methods:**

Using TCMSP, we screened the chemical components of 5 drugs. The components were input into the UniProt and PubChem databases to obtain target proteins; Genecards, Online Mendelian Inheritance in Man (OMIM), and CEO databases were used to screen target proteins for periodontal disease. The targets were imported into the Cytoscape software to obtain intersecting targets, and conduct visual analysis to build the PPI network. The intersecting targets were then input into the Matescape database and subjected to biological process (BP) analysis, molecular function (MF) analysis, cell component (CC) analysis, and KEGG enrichment analysis.

**Results:**

Twenty-seven TCM chemical components were obtained, with 198 target proteins associated with drugs and 2587 target proteins for periodontitis. Ten core targets were identified. Gene Ontology (GO) functional enrichment analysis yielded results for 20 BP, 11 MF, and 10 CC. KEGG analysis revealed that the main mechanisms of action were related to MAPK signaling pathway. Molecular docking results showed that luteolin strongly bind to TNF, IL6, and IL1B target proteins.

**Conclusion:**

The mechanism underlying the treatment of periodontitis with the recipe formula may be closely related to multiple targets in the MAPK signaling pathway.

## 1. Introduction

With the aging of the population, chronic periodontitis has become an important factor endangering dental health and affecting people daily activities.^[1,2]^ Bacterial invasion of gingival tissue and periodontal pathogens are the main causes of chronic periodontitis.^[3]^ Common clinical manifestations include redness and swelling, gum bleeding, toothache, difficulty chewing, and loose teeth, with may lead to tooth loss in some severe cases.^[4]^ Periodontitis has been closely associated with many major diseases, such as hypertension,^[5]^ Parkinson disease,^[1]^ and Alzheimer disease.^[2]^ The Fourth National Epidemiological Survey of Oral Health in China reported that the rate of healthy teeth in people older than 65 years old was only 9.3%, and tooth loss caused by eating difficulties has plagued a large number of elderly Chinese people, with periodontitis accompanied by diabetes severely affecting the daily activities of the elderly.^[1]^ In addition, the survey results showed that a low frequency of brushing, shorter brushing time, smoking, and other bad habits increased the incidence of the disease.^[2]^

Periodontitis is a sustained infectious disease, and treatment mainly focuses on preventing bacterial invasion and eliminating persistent infections of the periodontal tissue. The main therapies include taking antibiotics, subgingival scaling, phototherapy, periodontal tissue regeneration, and orthodontic treatment. Yet, antibiotics and other medications have been associated with some side effects, such as nausea, vomiting, diarrhea, and gastrointestinal problems. On the other hand, instrumental treatments cannot effectively clear pathogenic bacteria in important areas such as root bifurcations and deep periodontal tissues.^[6]^

**Figure 1.**
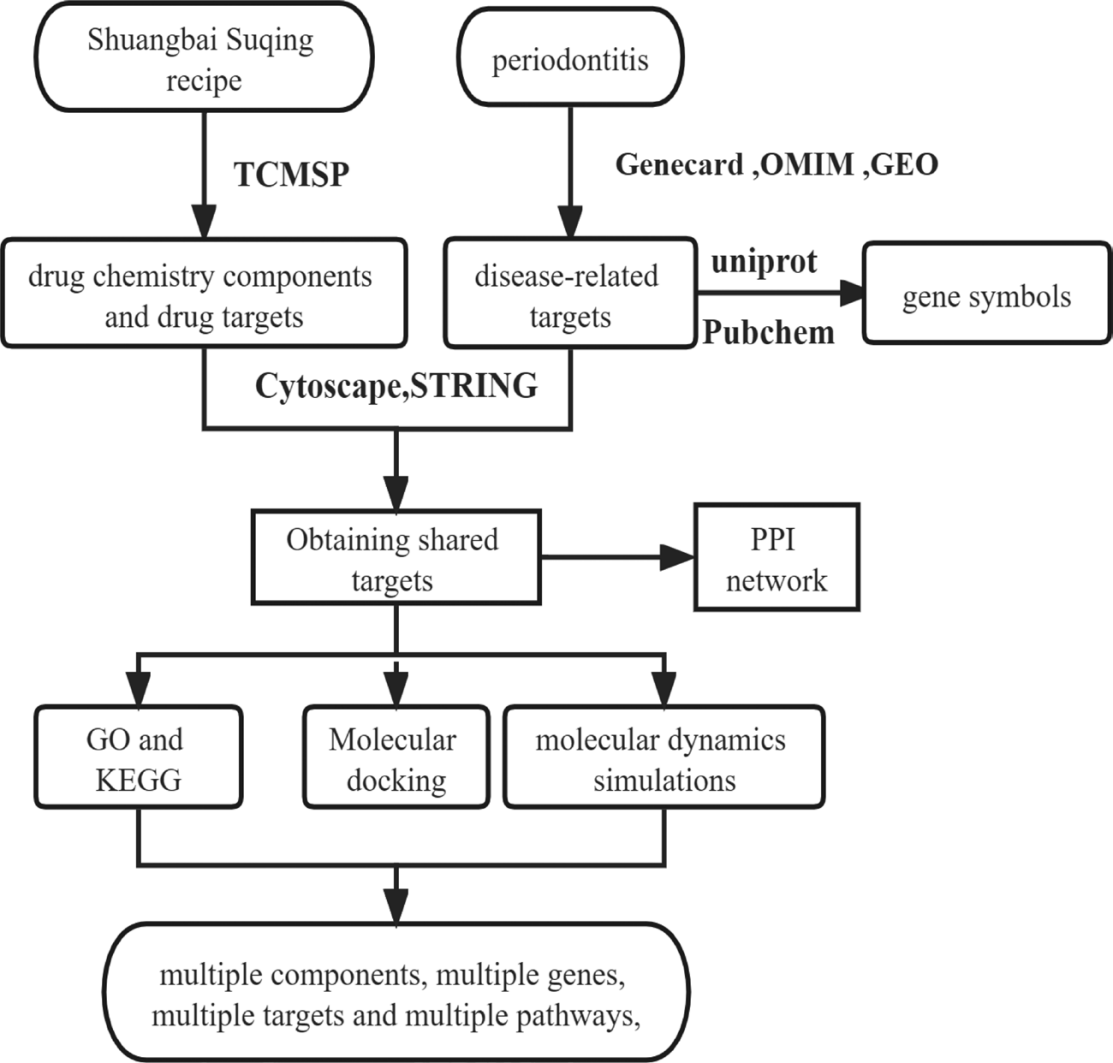
Flow chart of the pharmacology study on the Shuangbai Suqing recipe in treating periodontitis.

Therefore, seeking safer and more effective treatment methods for preventing and treating periodontitis has become increasingly important in the medical field. In recent years, the application of network pharmacology in studying TCM has gained widespread attention, as it provides a convenient, innovative, comprehensive, and effective new approach for researching diseases and drugs’ chemical components, targets, and interaction networks. In this study, network pharmacology was used to explore the material basis and mechanism of action of the Shuang Bai Su Qing recipe in the treatment of periodontitis, providing a basis for further clinical and pharmacological research on traditional Chinese medicine for treating periodontitis (Fig. 1).

## 2. Materials and methods

### 2.1. Search and screening for drug chemistry components and potential drug targets

Through the Traditional Chinese Medicine Systems Pharmacology (TCMSP)^[7]^ analysis platform, the main chemical components of 5 drugs, including *Bletilla Striata, Imperatae Rhizoma, Ecliptae Herba, Galla Chinensis*, and *Houttuyniae Herba* were searched. Oral bioavailability (OB) is one of the most important pharmacokinetic parameters.^[8]^ A compound with OB ≥ 30% is considered to have high OB. Druglikeness (DL) index is used to evaluate the drug-likeness of compounds and is employed for rapid screening of active substances.^[9]^ In the DrugBank database, the average DL index is 0.18. Compounds with a DL index ≥0.18 are considered to have higher drug-likeness. Screening criteria were selected as having OB ≥ 30% and drug-likeness (DL) ≥ 0.18. However, literature research identified compounds with a DL index ≥0.18 but OB < 30%.

### 2.2. Acquisition of disease-related targets

Targets related to periodontitis were obtained using “periodontitis” as a keyword. Periodontitis targets were obtained using the Genecard and Online Mendelian Inheritance in Man (OMIM) databases, and further supplemented by analyzing the GEO database (GES59939) using R language.^[10]^ The obtained targets were then input into the Uniprot and Pubchem databases, with official gene symbols replacing the corresponding targets, and any targets without gene symbols were removed.

### 2.3. Obtaining shared targets between the chemical component targets of the drugs and periodontitis targets

The selected chemical component targets of the Chinese medicine and periodontitis disease targets were separately imported into the Cytoscape 3.7.2 software to obtain the intersection of drug and disease targets. Then, an online Venn diagram was created using the Venn diagram tool.

### 2.4. Construction of target interaction network

The obtained intersection genes were imported into the String data analysis platform to construct a protein-protein interaction (PPI) network, which was then subjected to visualization analysis. The exported TSV-formatted data were imported into Cytoscape for screening and construction of the PPI network. The core target genes were selected using the MCC algorithm of the Cytoscape software cytoHubba plugin.

### 2.5. Construction of drug component-target-disease-gene interaction network

The core targets obtained through the Cytoscape 3.7.2 analysis software were used to construct a drug component-target-periodontitis-gene interaction network.

### 2.6. GO and KEGG pathway enrichment analysis

Using the Metascape database, the obtained genes related to drug treatment of periodontitis were imported for Gene Ontology (GO) and KEGG pathway enrichment analysis to obtain relevant data. In addition, the obtained data were imported into a bioinformatics website for obtaining GO analysis charts and KEGG enrichment pathway maps.

### 2.7. Obtaining small and large molecules prior to molecular docking

Using the TCMSP database, chemical structures of Chinese medicine components were searched and saved in mol2 format. The 3D structure images of target proteins TNF, IL6, VEGFA, AKT1, MMP9, PTGS2, CASP3, CXCL8, EGF, and IL1B were searched using the PDB database for “Homo sapiens” species with a small Resolution value and were then downloaded. AutoDock software was used to remove water, hydrogenate, and ligands from the large protein, followed by docking with small molecules to output results. The obtained docking results were imported into Pymol for visualization analysis.

### 2.8. Molecular dynamics simulations

The molecular dynamics (MD) simulations were carried out by GROMACS 2020.3 software. The amber99sb-ildn force field and the general Amber force field (GAFF) were used to generate the parameter and topology of proteins and ligands, respectively. The simulation box size was optimized with the distance between each atom of the protein and the box >1.0 nm. Then, fill the box with water molecules based on a density of 1. To make the simulation system electrically neutral, the water molecules were replaced with Cl^-^ and Na^+^ ions. Following the steepest descent method, energy optimization of 5.0 × 10^4^ steps was performed to minimize the energy consumption of the entire system, and finally to reduce the unreasonable contact or atom overlap in the entire system. After energy minimization, first-phase equilibration was performed with the NVT ensemble at 300 K for 100 ps to stabilize the temperature of the system. Second-phase equilibration was simulated with the NPT ensemble at 1 bar and 100 ps. The primary objective of the simulation is to optimize the interaction between the target protein and the solvent and ions so that the simulation system is fully pre-equilibrated. All MD simulations were performed for 50 ns under an isothermal and isostatic ensemble with a temperature of 300 K and a pressure of 1 atmosphere. The temperature and pressure were controlled by the V-rescale and Parrinello-Rahman methods, respectively, and the temperature and pressure coupling constants were 0.1 and 0.5 ps, respectively. Lennard-Jones function was used to calculate the Van der Waals force, and the nonbond truncation distance was set to 1.4 nm. The bond length of all atoms was constrained by the LINCS algorithm. The long-range electrostatic interaction was calculated by the Particle Mesh-Ewald method with the Fourier spacing 0.16 nm.

## 3. Results

### 3.1. Screening of drug components and potential targets

Through the TCM Systems Pharmacology Database (TCMSP), a search for 5 traditional Chinese medicines, namely *B striata, Imperata Rhizoma, Euphorbia Herba, G Chinensis*, and *Houttuynia Herba*, yielded 10 effective components for *B striata*, 5 effective components for *Imperata Rhizoma*, 11 effective components for *Euphorbia Herba*, 1 effective component for *G Chinensis*, and 6 effective components for *Houttuynia cordata*. After removing duplicate and non-targeted components, as well as those without corresponding official names of targets, 27 key chemical components (Table 1) such as quercetin, acacetin, pratensein, luteolin, and kaempferol, as well as 220 component-target pairs were identified.

**Table 1:** 27 chemical components of the 5-flavor medicines.

MOL	Compound	Degree	CAS	OB (%)	DL	Source
MOL000098	quercetin	154	73123-10-1	61.85	0.26	Mo Li Cao, Yu Xing Cao
MOL000422	kaempferol	63	520-18-3	54.18	0.55	Yu Xing Cao
MOL000006	luteolin	57	491-70-3	39.09	0.29	Mo Li Cao
MOL000358	beta-sitosterol	38	83-46-5	30.22	0.74	Bai Mao Gen
MOL000449	Stigmasterol	31	83-48-7	37.98	0.55	Bai Mao Gen
MOL001689	acacetin	26	480-44-4	31.46	0.78	Mo Li Cao
MOL005768	4,7-dihydroxy-1-p-hydroxybenzyl-2-methoxy-9,10-dihydrophenanthrene	21	N/A	30.54	0.55	Bai Ji
MOL001884	Omaine	21	207-514-6	54.43	0.55	Bai Mao Gen
MOL003398	Pratensein	19	2284-31-3	43.74	0.86	Mo Li Cao
MOL003389	3’-O-Methylorobol	18	36190-95-1	22.29	0.27	Mo Li Cao
MOL000476	Physcion	17	521-61-9	53.66	0.22	Bai Ji
MOL004347	Laurifoline	16	6808-76-0	36.91	0.75	Mo Li Cao
MOL005755	1-(4-hydroxybenzyl)-4-methoxy-9,10-dihydrophenanthrene-2,7-diol	15	N/A	43.83	0.76	Bai Ji
MOL005761	3-(p-hydroxybenzyl)-4-methoxy-9,10-dihydrophenanthrene	15	N/A	26.6	0.51	Bai Ji
MOL005756	2,3,4,7-tetramethoxyphenanthrene	9	N/A	39.84	0.71	Bai Ji
MOL003404	wedelolactone	8	524-12-9	34.97	0.24	Mo Li Cao
MOL002975	butin	7	153-18-4	69.94	0.21	Mo Li Cao
MOL005766	3,7-dihydroxy-2,4-dimethoxyphenanthrene-3-O-glucoside	6	N/A	33.94	0.43	Bai Ji
MOL001870	LUTEOLINIDIN	6	1154-78-5	57.41	0.27	Mo Li Cao
MOL000569	digallate	3	536-08-3	39.06	0.28	Wu Bei Zi
MOL005773	blespirol	2	N/A	72.13	0.43	Bai Ji
MOL003402	demethylwedelolactone	2	6468-55-9	49.6	0.48	Mo Li Cao
MOL004350	Ruvoside_qt	2	6859-20-7	36.16	0.25	Yu Xing Cao
MOL005759	2,7-dihydroxy-4-methoxyphenanthrene-2,7-O-diglucoside	1	N/A	46.43	0.28	Yu Xing Cao
MOL005770	bletlol A	1	N/A	41.88	0.24	Bai Ji
MOL001790	Linarin	1	480-36-4	36.12	0.76	Mo Li Cao
MOL003378	1,3,8,9-tetrahydroxybenzofurano[3,2-c]chromen-6-one	1	6468-55-9	22.96	0.55	Mo Li Cao

DL = drug-likeness, OB = oral bioavailability.

### 3.2. Screening of periodontitis targets

Using the Genecards, OMIM and CEO databases with the keyword “periodontitis,” 2587 unique targets were obtained after removing duplicates.

### 3.3. Screening of shared targets between drugs and periodontitis and construction of PPI network

**Figure 2.**
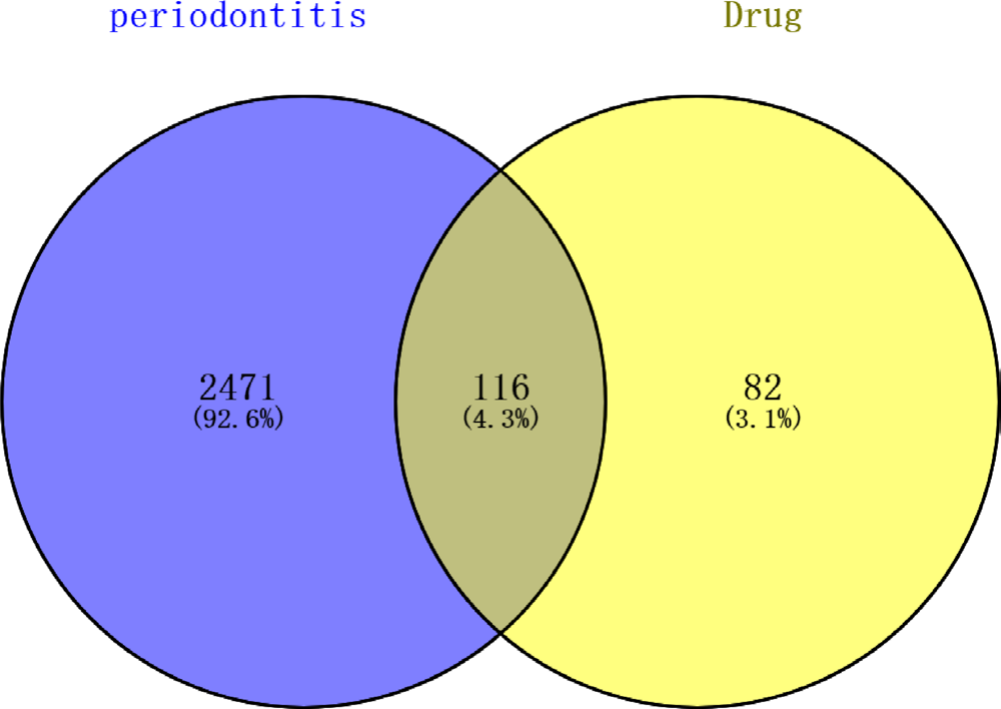
Venn diagram of drug composition targets and periodontitis targets.

**Figure 3.**
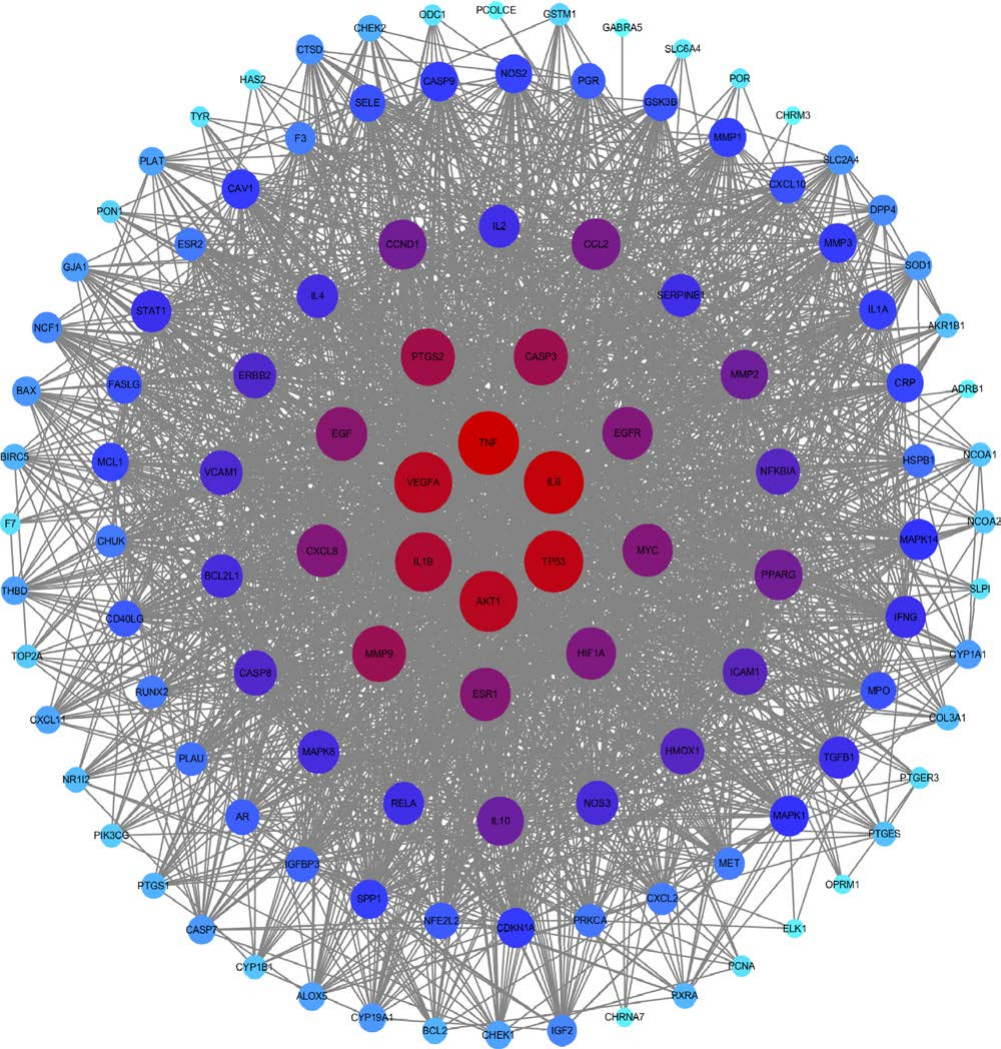
PPI network of 5 drugs and the core targets shared with periodontitis. The darker the color, the larger the node, and the larger the degree value, the more important the target. PPI = protein-protein interaction.

**Figure 4.**
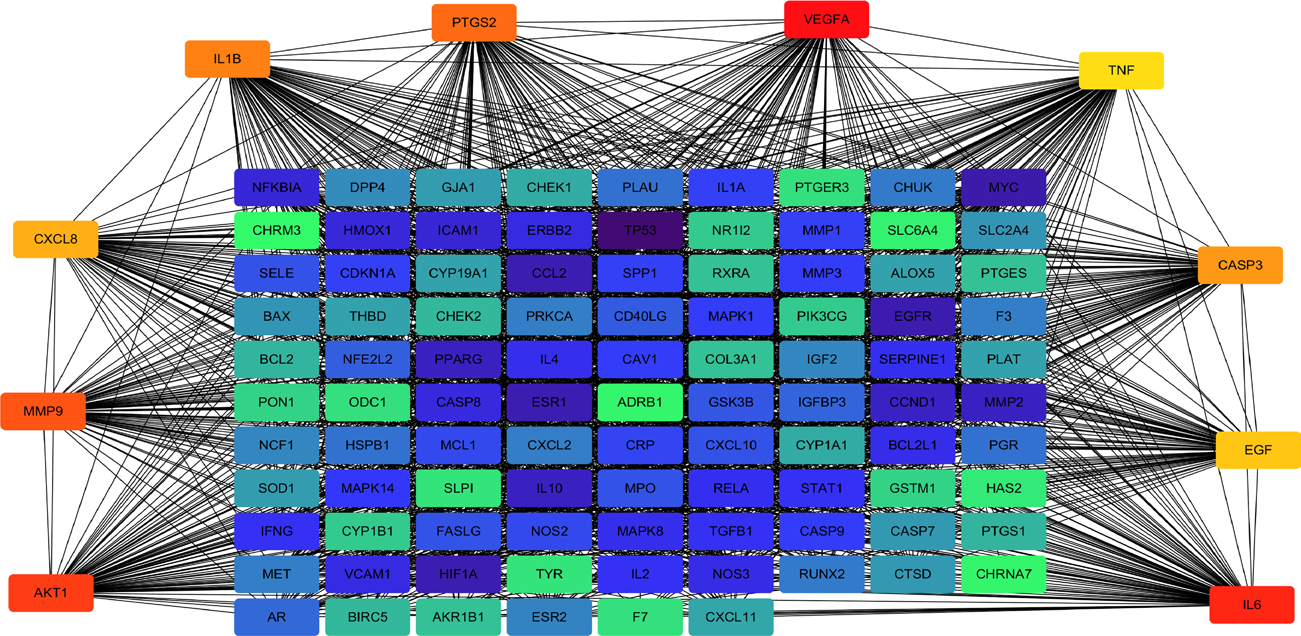
Interaction maps of the 10 key targets screened with other targets.

The related targets obtained were imported into the Cytoscape software, and 115 common targets between drugs and diseases were obtained. A Venn diagram was constructed using an online Venn diagram tool (Fig. 2). The common genes obtained in the previous step were imported into the String data analysis platform to construct a PPI network and perform visualization analysis. The TSV format data obtained was then imported into the Cytoscape analysis software for analysis, resulting in a network diagram with 115 nodes and 2147 edges (Fig. 3). Utilize the MCC algorithm of the cytoHubba plugin in the Cytoscape software to filter and obtain 10 core target genes, primarily including TNF, IL6, TP53, AKT1, and VEGFA (Fig. 4) (Table 2).

**Table 2:** Topological analysis information of 10 targets.

Rank	Name	Score	Degree
1	VEGFA	9.64E + 26	87
2	IL6	9.64E + 26	91
3	AKT1	9.64E + 26	87
4	MMP9	9.64E + 26	78
5	PTGS2	9.64E + 26	80
6	IL1B	9.64E + 26	84
7	CASP3	9.63E + 26	79
8	CXCL8	9.62E + 26	71
9	EGF	9.61E + 26	73
10	TNF	9.58E + 26	93

AKT1 = serine/threonine protein kinase 1, CASP3 = Caspase-3, IL1B = interleukin-1B, IL6 = interleukin-6, PTGS2 = prostaglandin G/H synthase 2, TNF = tumor necrosis factor, VEGFA = vascular endothelial growth factor A.

### 3.4. Construction of drug compound-target-periodontitis-gene interaction network

**Figure 5.**
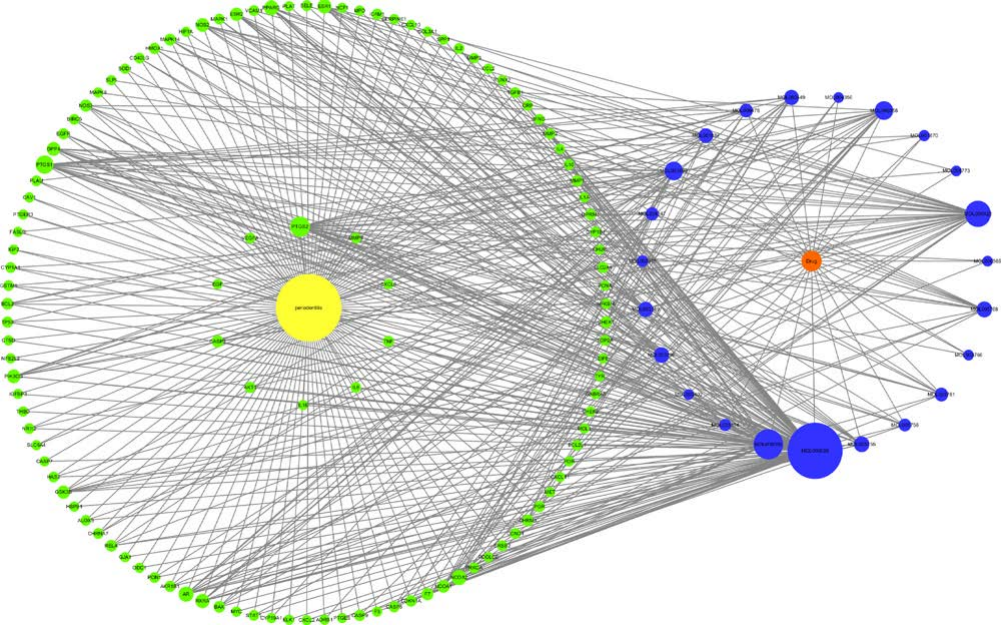
The network diagram of drug chemical composition-target-disease-gene interaction. Orange represents drugs, blue represents drug compounds, yellow represents diseases, and Green represents intersecting genes. The larger the node and the more connections it has, the more important it is.

After importing the 10 core intersecting targets into Cytoscape software, the network diagram of drug chemical composition-target-periodontitis disease-target interaction (Fig. 5) was obtained.

### 3.5. GO and KEGG enrichment analysis

**Figure 6.**
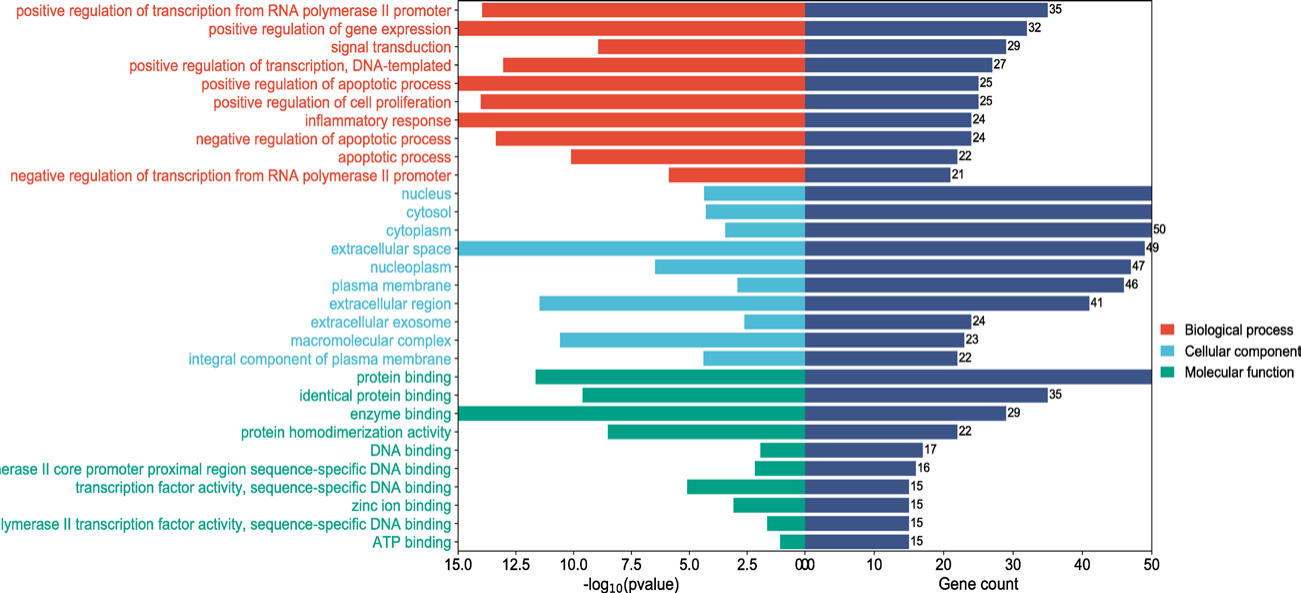
GO analysis of targets for 5 drugs used to treat periodontitis. GO = Gene Ontology.

**Figure 7.**
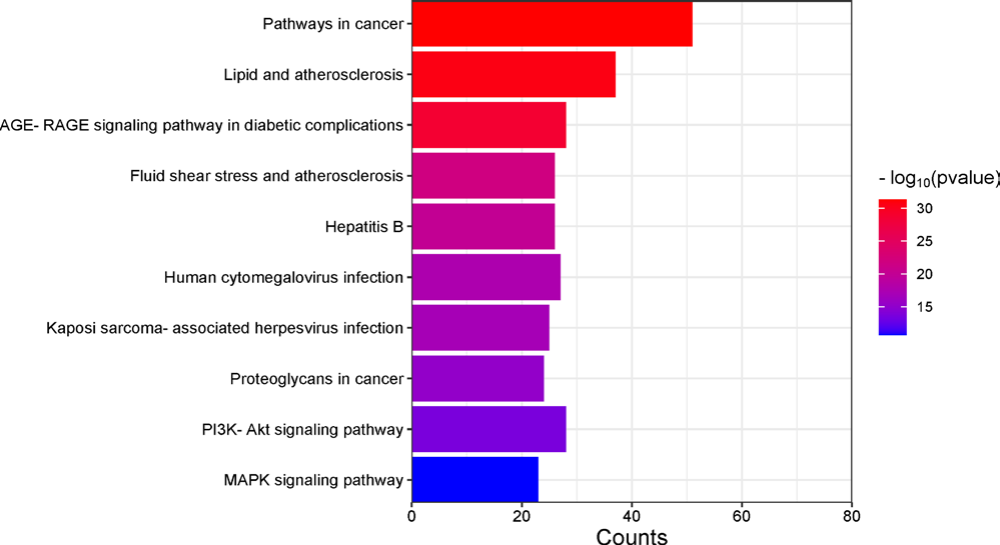
KEGG analysis of targets for 5 drugs used to treat periodontitis. KEGG = Kyoto Encyclopedia of Genes and Genomes.

The 115 intersecting targets were input into the Matescape database to obtain relevant data, and the common targets were subjected to GO biological process analysis, GO molecular function analysis, GO cellular component analysis, and KEGG enrichment analysis. After 3 different GO analyses, the top 10 biological processes (BP), top 11 molecular functions (MF), and top 10 cellular components (CC) were selected for analysis (Fig. 6). The BP entries were the largest in number and most relevant in the GO analysis, mainly involving positive regulation of transcription from RNA polymerase II promoter, positive regulation of gene expression, signal transduction, inflammatory response, negative regulation of apoptotic process, apoptotic process, nucleus, cytosol, cytoplasm, extracellular space, as well as plasma membrane, extracellular exosome, integral component of plasma membrane, protein binding, response to inorganic substances, cellular response to lipid, cellular response to organic cyclic compound, and cellular response to the organonitrogen compound. The KEGG pathway enrichment analysis results (Fig. 7) showed mainly the PI3K-Akt signaling pathway, pathways in cancer, lipid and atherosclerosis, AGE-RAGE signaling pathway in diabetic complications, hepatitis B, human cytomegalovirus infection, MAPK signaling pathway, etc.

### 3.6. Molecular docking results

**Figure 8.**
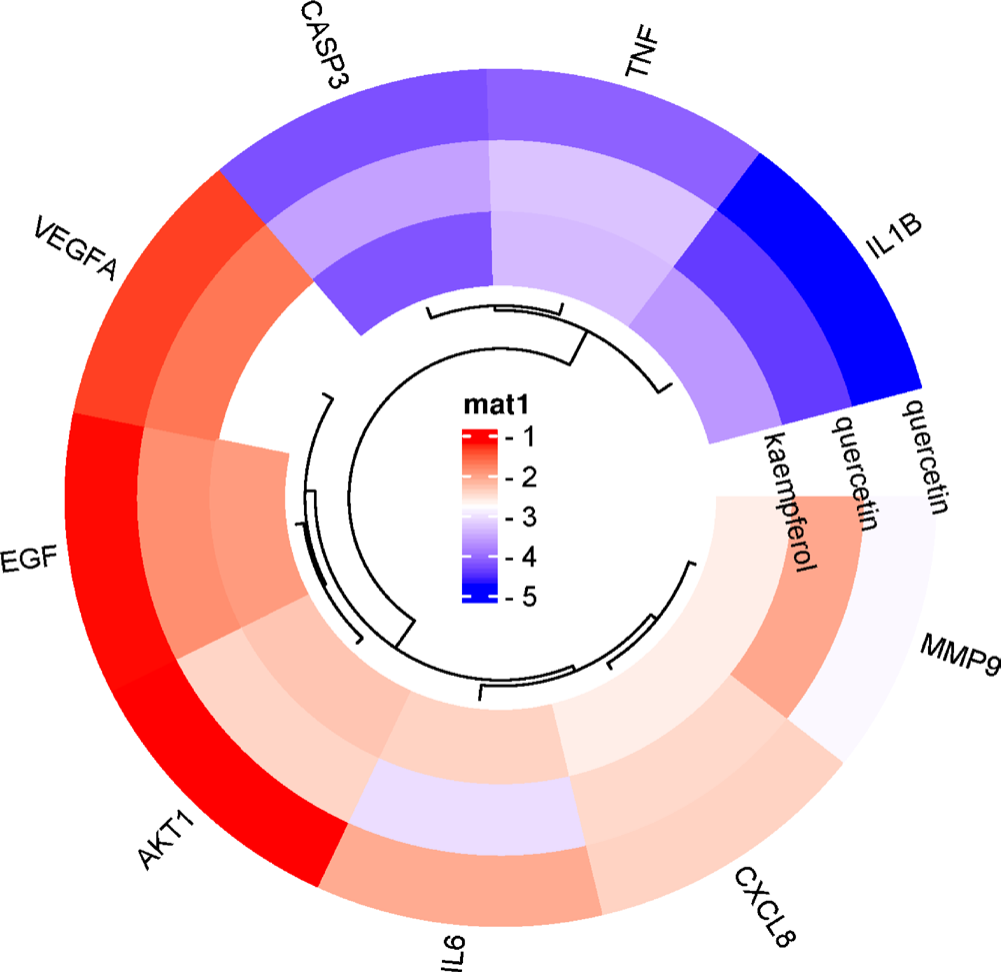
Olecular docking score.

**Figure 9.**
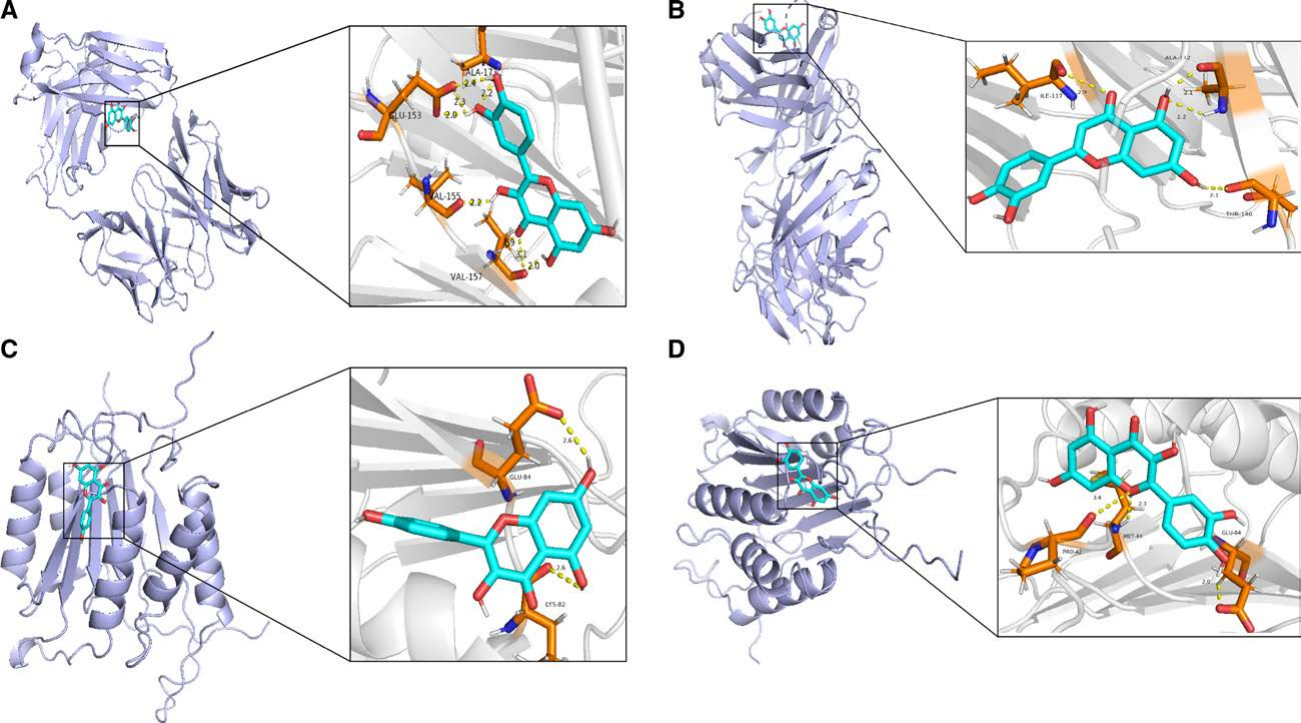
Molecular docking of key traditional Chinese medicine components with target proteins. (A) Binding of quercetin active component with IL1B protein. (B) Binding of luteolin active component with IL1B protein. (C) Binding of kaempferol with CASP3 protein. (D) Binding of quercetin active component with CASP3 protein. CASP3 = Caspase-3.

The molecular docking of the chemical components of quercetin, luteolin, and kaempferol with key targets TNF, IL6, VEGFA, AKT1, MMP9, PTGS2, CASP3, CXCL8, EGF, and IL1B resulted in docking scores that indicate the strength of the binding energy between the molecule and the target protein. Lower docking scores indicate a stronger binding capacity between the molecule and the target (Fig. 8). The chemical components of TCM showed a strong binding affinity with IL1B, TNF, and CASP3 key target proteins. In addition, the docking results showed that the active component quercetin could bind to the GLU-153, VAL-155, ALA-17, and VAL-157 active sites of the IL1B protein and form 6 hydrogen bonds (Fig. 9A) while binding to the PRO-42, MET-44, and GLU-84 active sites of the CASP3 protein could form 5 hydrogen bonds (Fig. 9D). The active component luteolin could bind to the ILE-117, ALA-142, and THR-140 active sites of the IL1B protein and form 4 hydrogen bonds (Fig. 9B). Kaempferol could bind to the LYS-82 and GLU-84 active sites of the CASP3 protein and form 6 hydrogen bonds (Fig. 9C).

### 3.7. Root-mean-square deviation

**Figure 10.**
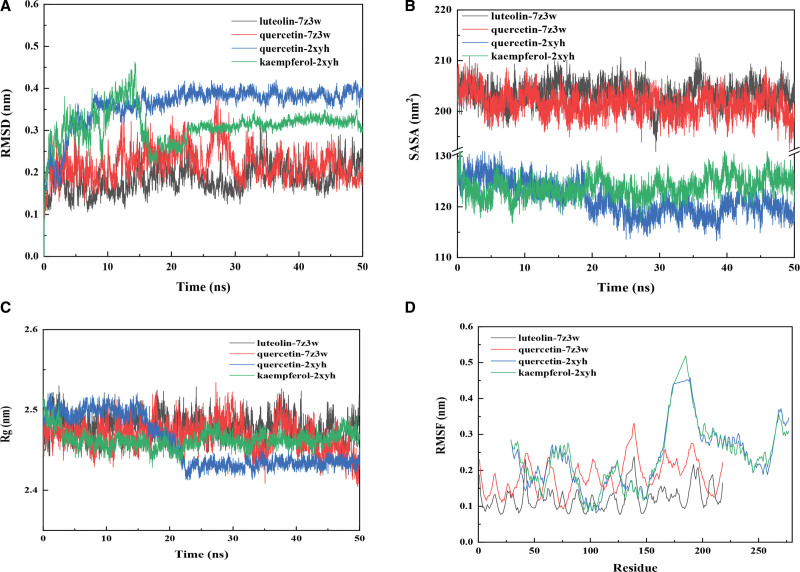
The change in RMSD, SASA, Rg, and RMSF of the protein during the complex simulation process over time.

Root mean square deviation (RMSD) measures the coordinate deviation of a specific atom relative to a reference structure and is often used to assess whether a simulation system has reached stability. A stable RMSD indicates that the corresponding atom has become stable, while a fluctuating RMSD indicates fluctuations. As shown in Figure 2, luteolin-7z3w and quercetin-7z3w protein ligands reach equilibrium after 30 ns, while quercetin-2xyh and kaempferol-2xyh protein ligands reach equilibrium after 15 ns. Overall, RMSD reaches equilibrium after a certain period of time, indicating the reliability of the entire simulation (Fig. 10A).

### 3.8. The solvent-accessible surface areas

Solvent accessible surface area (SASA) is calculated by considering the interaction between solute molecules and solvent molecules through van der Waals forces. The SASA of a protein decreases with increasing protein compactness, so changes in SASA can predict changes in protein structure. As shown in Figure 2, the SASA values of luteolin-7z3w, quercetin-7z3w, and kaempferol-2xyh proteins show no significant changes, while the SASA of the protein in the quercetin-2xyh complex decreases, indicating an increase in protein compactness in the quercetin-2xyh complex system (Fig. 10B).

### 3.9. Radius of gyration

Gyration radius (Rg) is used to demonstrate the compactness of protein structures during the simulation process, and it represents the distance between the center of mass of all atoms within a specific time interval and their ends. Figure 3 shows that Rg exhibits a similar trend to SASA throughout the entire complex MD simulation process, indicating an increase in protein compactness in the quercetin-2xyh complex system (Fig. 10C).

### 3.10. Root mean square fluctuation

Root mean square fluctuation (RMSF) calculates the fluctuations of each atom relative to its average position, representing the average change of the structure over time and providing a characterization of the flexibility of different regions of the protein. From Figure 4, we can observe that the RMSF values of the kaempferol-2xyh and quercetin-2xyh protein ligands are higher than those of the luteolin-7z3w and quercetin-7z3w protein ligand complexes, indicating that the 2xyh group has higher flexibility than the z3w group (Fig. 10D).

### 3.11. Hydrogen bond analysis

**Figure 11.**
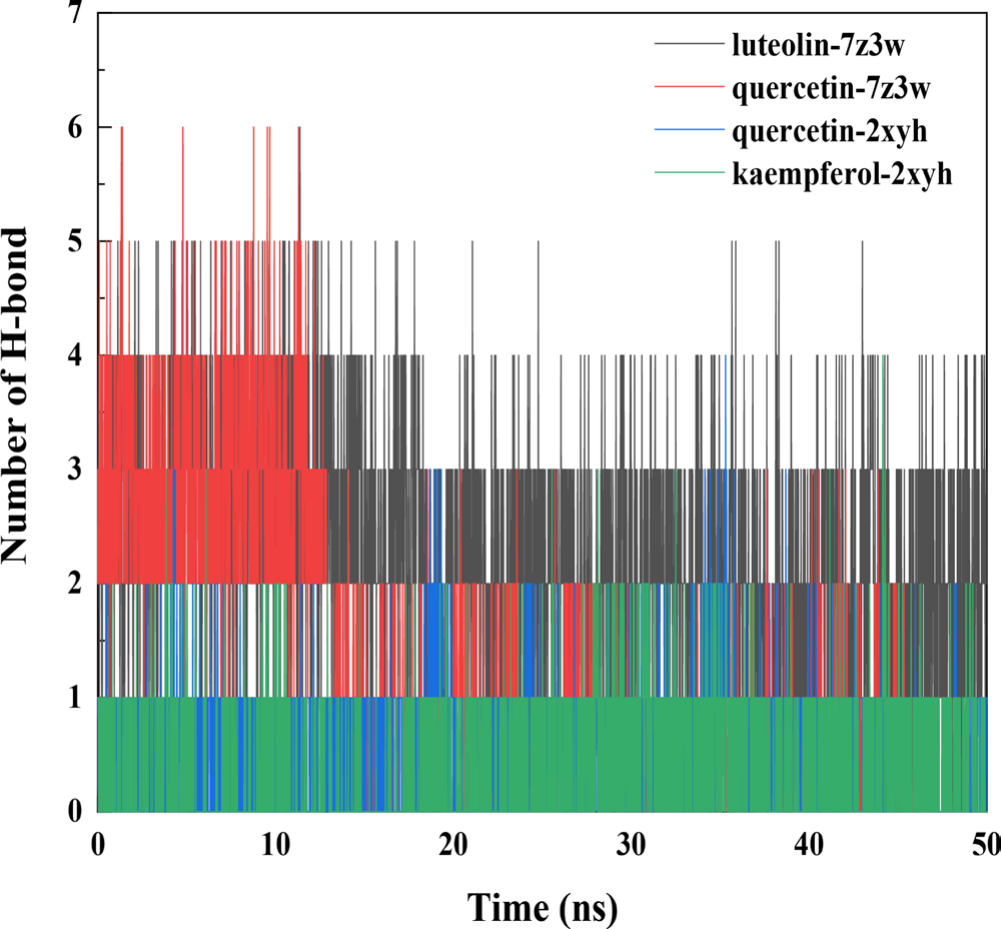
The change in the number of hydrogen bonds in the protein-ligand complex during the complex simulation process over time.

To investigate the interaction between the protein and ligands, we first conducted hydrogen bond analysis of the protein-ligand complexes. As shown in Figure 5, the average number of hydrogen bonds between luteolin-7z3w, quercetin-7z3w, quercetin-2xyh, and kaempferol-2xyh proteins and small molecules are 2.22, 1.15, 0.72, and 0.42, respectively. This indicates the presence of hydrogen bond interactions between the proteins and ligands (Fig. 11).

### 3.12. MMPBSA the free energy

To better explain the interaction energy between the protein and ligands, we used the gmx_mmpbsa (https://jerkwin.github.io/gmxtool/) script to determine the binding energies of all protein-ligand complexes at the equilibrium stage. In the application of the MMPBSA method, the total binding energy is decomposed into 4 independent components (electrostatic interactions, van der Waals interactions, as well as polar and nonpolar solvation interactions), with the nonpolar solvation term commonly referred to as SASA. The binding energies of the protein-ligand complexes are shown in Table 1. In the protein-ligand complex systems, the binding free energies of luteolin-7z3w, quercetin-7z3w, quercetin-2xyh, and kaempferol-2xyh proteins with small molecules are negative, with values of −67.886, −26.513, −56.895, and −58.013 kJ/mol, respectively. This indicates that the protein-ligand complexes can stably bind, with the main interaction energy being attributed to van der Waals interactions (Table 3).

**Table 3:** Protein-ligand MMPBSA analysis.

Energy	luteolin-7z3w	quercetin-7z3w	quercetin-2xyh	kaempferol-2xyh
Van der Waals Energy (kJ/mol)	−137.924	−87.613	−101.750	−90.756
Electrostatic energy (kJ/mol)	−33.217	−102.950	−58.514	−14.583
Polar solvation energy (kJ/mol)	101.327	159.635	103.728	44.001
Nonpolar solvation Energy (kJ/mol)	−16.041	−13.104	−13.908	−12.417
Total Binding Energy (kJ/mol)	−85.855	−44.032	−70.443	−73.755
T ∆ S (kJ/mol)	17.969	17.519	13.548	15.742
Total Binding Free Energy (kJ/mol)	−67.886	−26.513	−56.895	−58.013

## 4. Discussion

The study of periodontitis in TCM has a long history and is associated with disease names such as “yaxuan” and “chichuang,” which are mentioned in numerous classical texts, including the Yellow Emperor Inner Canon. E.g., the Tang Dynasty Bei Ji Qian Jin Yao Fang records symptoms such as “swollen and painful gums” and “bleeding between teeth” related to periodontitis.^[11]^ TCM classifies periodontitis into 2 types based on its etiology (external and internal) and 3 types based on its pathogenesis (deficiency of kidney yin, stomach fire inflammation, and deficiency of Qi and blood). The Zhi Zhi Fang states,^[12]^ “teeth are the endpoint of bones and the source of marrow, which are governed by the kidney. Therefore, tooth decay occurs when the kidney weakens; teeth become strong when the essence is abundant, while tooth movement occurs due to deficiency heat.” Modern research shows that replenishing the kidney can reduce the number of inflammatory cytokines associated with diabetes complicated by periodontitis.^[13]^ Similarly, boosting Qi and yang can reduce the expression of inflammatory cytokines associated with periodontitis.^[14]^

Traditional Chinese medicine (TCM) has been applied for more than 4000 years in treating various diseases by nourishing yin and clearing heat, strengthening teeth, and promoting vitality. For example, *B Striata* (Baiji), *I Rhizoma* (Baimaogen), *E Herba* (Mohanlian), *G Chinensis* (Wubeizi), and *H Herba* (Yuxingcao) recipe have been used for the treatment of periodontitis.^[15–18]^. This herbal recipe has the advantages of quick action, convenient administration, good taste, and low cost. However, the mechanism of action is still unclear.

Studies have shown that extracts from *G Chinensis* have protein precipitation and coagulation effects, and they exhibit antibacterial and antiviral activities by inhibiting the formation of bacterial biofilms.^[19,20]^ The chemical components present in *G Chinensis* do not harm beneficial bacteria that adhere to teeth but inhibit harmful bacterial populations in the oral cavity.^[21]^ Extracts of *H cordata*, especially the volatile oil fraction, demonstrate the best inhibitory effects against Porphyromonas gingivalis in vitro.^[22]^ Extracts of Imperata cylindrica can inhibit the activation of TLR4 and NF-κB signaling pathways and downregulate the release of inflammatory factors.^[23]^ Polysaccharide compound *B striata* polysaccharide, formed by the polymerization of extracts from Paeonia lactiflora, possesses characteristics such as anti-inflammatory, antioxidant, and strong hydrophilicity, making it suitable for use as a raw material in the formulation of drugs for the treatment and prevention of periodontitis.^[24]^ Research conducted by Wu Tian has shown that extracts of Dryopteris crassirhizoma alleviate tissue inflammation by reducing protein and gene expression of NF-κB and TNF-α,^[25,26]^ and they also show therapeutic effects in the treatment of osteoporosis.^[27,28]^

Based on years of clinical experience, the author has developed a new formula called Shuang Bai Su Qing Formula, which aims to nourish yin, clear heat, strengthen teeth, and nourish essence. The main herbs in the formula include *B Striata, I Rhizoma, E Herba, H Herba*, and *G Chinensis. H Herba* can clear heat and toxins, eliminate abscesses, and drain pus. *I Rhizoma* and *E Herba* are assistant herbs that work together to clear lung and stomach fire while enhancing the detoxifying power of the main herb. *E Herba* is particularly effective in nourishing the kidney yin, which cleanses the oral cavity and strengthens teeth by nourishing the essence. *B Striata* and *G Chinensis* are used as adjuvant herbs, with the former having an astringent effect of assisting in clearing heat and stopping bleeding, while the latter augments the assistant herbs’ ability to nourish the kidney yin in addition to its astringent properties.^[11–14]^ Combining these 5 herbs achieves a balance of clearing and nourishing, treating both symptoms and root causes, resulting in a good therapeutic effect.

This study employed network pharmacology and molecular docking methods to further investigate the mechanism of action of Shuang Bai Su Qing Formula in treating periodontitis. The results demonstrated that the main chemical constituents responsible for the anti-periodontitis effects of the 5 herbs mentioned above are quercetin, luteolin, kaempferol, and other chemical components. The topological analysis yielded 115 core targets that have a therapeutic role, including TNF, IL6, TP53, AKT1, VEGFA, IL1B, PTGS2, and CASP3, with TNF, IL6, VEGFA, AKT1, MMP9, PTGS2, CASP3, CXCL8, EGF, and IL1B being the key targets due to their higher degree values. By constructing a drug-disease protein interaction network and performing GO and KEGG enrichment analyses, the key pathways of the 5 TCM, besides cancer and hepatitis B pathways and cellular pathways, are mainly closely related to the PI3K-Akt signaling pathway and MAPK signaling pathway. Previous studies have shown that the PI3K-Akt signaling pathway inhibits the apoptosis of periodontal ligament fibroblasts induced by high glucose,^[29]^ while the MAPK signaling pathway is an important signal pathway for regulating the body inflammatory response and is highly relevant to periodontitis.^[30]^ Among the 4 MAPK pathways, p38 is closely related to inflammation. After p38 is activated, it can promote the release of inflammatory factors and further activate the NF-κB pathway, leading to excessive expression of inflammatory factors.^[31]^ Activation of p38 MAPK can increase the expression of pro-inflammatory cytokines and participate in the immune response of cells, thereby promoting the development of diseases. P38 inhibitors are an effective treatment for inflammation.^[32]^ Scholars have demonstrated that many chemical components or drugs can treat periodontitis by inhibiting the p38MAPK pathway, such as *Moringa oleifera* leaf extract,^[33]^ 1,25-dihydroxyvitamin-D3,^[34]^ and Rho kinase inhibitor Y-27632.^[35]^ Additionally, p38MAPK can also upregulate the osteogenic ability of human periodontal ligament stem cells.^[36]^ The relationship between MAPK and periodontitis has received increasing attention in the medical field. Developing drugs that can block or promote the MAPK pathway is becoming a promising research direction for the effective treatment of periodontitis.

This study demonstrated that the Shuang Bai Su Qing Formula conforms to this direction. Meanwhile, molecular docking studies show that the active ingredients of TCM can bind to key target proteins with negative docking scores. The docking scores for quercetin with IL1B protein and TNF protein and the docking score for luteolin with IL1B protein were all below-4. The smaller score was associated with the lower required reaction energy, which further validated the potential target proteins of this formula in treating periodontitis. Molecular dynamics simulation results showed that the RMSD reached equilibrium after a certain time, the protein SASA and Rg in quercetin-2 xyh composite system decreased, which indicates that the protein tightness increased in quercetin-2 xyh composite system, RMSF showed that 2 xyh kneading activity is >7z3w group, further proved that the protein coordination and stable binding, and the main interaction can be the van der Waals force interaction. It also further verifies the possible target role of this protein in the treatment of periodontitis.

Overall, the Shuangbai Suqing Formula for treating periodontitis has the characteristics of multiple components, multiple genes, multiple targets, multiple pathways, and multiple approaches. The key targets and MAPK signaling pathway obtained in this study provide a solid foundation for further accurate and efficient basic research as well as clinical applications.

## Author contributions

**Conceptualization:** Yongzhi Chen.

**Data curation:** Yongzhi Chen, Haomin Zhang.

**Formal analysis:** Haomin Zhang.

**Investigation:** Haomin Zhang, Ruirui Ning.

**Methodology:** Haomin Zhang, Ruirui Ning.

**Project administration:** Ruirui Ning.

**Resources:** Haomin Zhang.

**Software:** Ruirui Ning.

**Supervision:** Haomin Zhang.

**Validation:** Haomin Zhang, Ruirui Ning, Yongqin Zhao.

**Visualization:** Haomin Zhang, Zhao Yan.

**Writing – original draft:** Haomin Zhang.

**Writing – review & editing:** Ruirui Ning.
